# Vape Gods and Judaism—E-cigarettes and Jewish Law

**DOI:** 10.5041/RMMJ.10372

**Published:** 2019-07-18

**Authors:** Sharon Galper Grossman

**Affiliations:** Halakhic Advisor (Morah L’Halakha), Matan HaSharon, Ra’annana, Israel; and Oncology Consultant, Ra’annana, Israel

**Keywords:** E-cigarettes, Jewish law, vaping

## Abstract

**Objective:**

To review current medical literature on the risks and potential benefits of e-cigarette use and its permissibility under Jewish law.

**Methods:**

A survey of current medical literature about the risks and potential benefits of e-cigarette use, and a review of existing rabbinic literature regarding both combustible and e-cigarette products.

**Results:**

E-cigarettes contain fewer harmful materials than do combustible cigarettes. However, they are not risk-free. Their skyrocketing use among youth is of concern, as e-cigarettes lead to nicotine addiction and are a gateway to combustible cigarettes. Preliminary data indicate that e-cigarettes increase the risk of myocardial infarction, chronic obstructive pulmonary disease (COPD), and emphysema and are no more effective as aids to smoking cessation than US Food and Drug Administration (FDA)-approved interventions with acceptable safety profiles. Few *halakhic* decisors have opined on the permissibility of e-cigarettes, but extrapolating from *halakhic* discussions regarding combustible cigarettes strongly suggests that they would prohibit e-cigarettes based on government warnings and preliminary data demonstrating increased risk of cardiovascular and respiratory diseases, at the least because of possible danger (*safek sakana*). Among youth and pregnant women, for whom e-cigarettes are particularly dangerous and for whom the government has administered explicit warnings, a Jewish legal prohibition should be absolute. There is a unique obligation to prevent youth from obtaining these products. Jewish law might also prohibit deriving benefit from the sale or advertisement of these products.

**Conclusions:**

Extrapolating from rabbinic literature regarding combustible cigarettes, the preliminary data establishing the dangers of e-cigarettes and the government warnings against usage would render these products prohibited under Jewish law, especially for youth and pregnant women.

## BACKGROUND

Introduced in China in 2003 and in the United States and Europe in 2006,^1^ electronic cigarettes (e-cigarettes) are battery-operated devices that heat a liquid to produce a vapor that the user inhales.^2^ The main components of the liquid vaporized are nicotine (although some are nicotine-free), propylene glycol or glycerol (known carcinogens),^3^,^4^ and flavorings such as candy, fruit, soda, and alcohol flavors^3^,^4^ that manufacturers add to make initial exposure more pleasurable for first-time users, especially middle-schoolers and teenagers.^5^,^6^ The products contain other compounds such as tin, lead, nickel, chromium, manganese, arsenic,^7^–^10^ tobacco-specific nitrosamines, carbonyl compounds, metals, volatile organic compounds, and phenolic compounds,^11^–^15^ all of which have carcinogenic potential. Although *e-cigarettes* do not expose users to many of the harmful compounds in cigarette smoke (e.g. tars, oxidant gases, and carbon monoxide),^8^ *e-cigarette vapors* nevertheless do contain potentially toxic, carcinogenic chemicals, raising concerns regarding the exposure of the user and those around him.^16^ Still, most experts believe that inhaling e-cigarette vapor is probably less harmful than inhaling cigarette smoke.^1^,^8^,^16^–^18^ *Less harmful, however, does not mean harmless*.

The dangers of nicotine, the chemical responsible for the extraordinary addictiveness of tobacco products, are manifold especially in youth. Nicotine is uniquely harmful to the brains of those under the age of 25, increasing the risk of addiction, mood disorders, lowered impulse control, cognitive impairment, and attention problems.^16^,^19^ Symptoms of nicotine addiction, such as drug withdrawal, the forfeiture of social, occupational, and recreational activities in favor of nicotine use, and the need to leave the classroom to vape cause substantial distress and impairment.

How much nicotine do e-cigarettes contain? The nicotine content of e-cigarettes varies, ranging from none (nicotine-free) to 36 mg/mL or higher.^7^,^16^ All JUUL products (the brand that has captured 50% of the market in just two years) contain nicotine. A recently published study found that only 25% of the individuals who recognized the JUUL product and 37% of current youth and young adult JUUL users were aware that JUUL always contains nicotine.^20^ Most e-cigarette products deliver nicotine even more efficiently than cigarettes. For example, the JUUL website claims that the nicotine in one JUUL cartridge equals the amount in a pack of cigarettes, or about 200 puffs. JUUL delivers nicotine 2.7 times faster than other e-cigarettes.

Furthermore, E-cigarettes are a *gateway to combustible cigarettes*. Youth who vape are more likely than non-users to start smoking cigarettes, and the higher nicotine concentrations in JUUL might heighten the likelihood of this transition.^16^ E-cigarettes preceded the use of combustible cigarettes for 36% of US youth who smoke. Over a two-year period, e-cigarettes introduced more than 260,000 youth to combustible cigarettes.^21^ Approximately 80% of the individuals who start smoking during adolescence continue to smoke in adulthood, and one-third of them will die prematurely from smoking-related diseases.^22^ There is growing concern that by creating an entire generation of nicotine-addicted youth, e-cigarettes will reverse the dramatic reductions in smoking achieved over the last 50 years. However, even if adolescents who use e-cigarettes do not switch to regular cigarettes, the harmful effects of nicotine addiction are extensive.^23^–^28^

We also know that nicotine is a *gateway to opioid addiction*, as nicotine lowers the threshold for addiction to other agents.^29^ We know that teens who vape are more likely than non-users to use other substances including alcohol, marijuana, and cocaine and to engage in sexual activity.^30^ Given the high nicotine concentrations in JUUL, the nicotine-related health consequences of its use by young people could be more severe than those from their use of other e-cigarette products.^16^

The long-term carcinogenic, respiratory, and cardiovascular effects of e-cigarettes and their flavorings are not yet fully known, but are thought to be substantially lower than smoking.^31^ There are currently no documented cases of lung cancer linked to e-cigarette use. But carcinogenesis can take years to develop after initial exposure, and vaping has only become popular in the last few years. E-cigarette vapor emits formaldehyde, a known carcinogen, at levels 5–15 times higher than normal cigarettes.^32^ In addition, animal studies show that, like conventional cigarettes, e-cigarettes have harmful effects on DNA.^33^ Similarly, the oral tissue of e-cigarette users demonstrates some of the same cancer-related molecular changes (potential precursors to cancer development) as noted in cigarette smokers.^34^ It may take many years to confirm the carcinogenic effects of vaping. However, preliminary data raise disturbing concerns that e-cigarettes cause cancer.

The long-term cardiovascular risks of e-cigarette use are also unknown, but preliminary research indicates they are substantially lower than those of combustible cigarettes.^31^ Nevertheless, biological studies show that e-cigarettes are associated with endothelial dysfunction, oxidative stress, inflammation, platelet activation, and activation of the sympathetic nervous system,^35^–^37^ all of which can increase the risk of heart attack. A recent study of 70,000 people from University of California San Francisco reported that daily e-cigarette use doubles the risk of myocardial infarction compared to that of non-smokers; cigarettes alone triple the risk, and the combined use of e-cigarettes and combustible cigarettes increases it five-fold.^38^ This study confirms that e-cigarettes are harmful.

The long-term respiratory effects of e-cigarette use are also unknown.^16^,^39^ Limited evidence suggests that changes in airway respiratory function are much smaller than those associated with conventional cigarettes, but also that e-cigarettes increase coughs, asthma symptoms, chronic bronchitis (especially among adolescents),^40^ emphysema, and chronic obstructive pulmonary disease (COPD).^41^ The flavorings might also affect respiratory function.^42^ Thus, while the long-term dangers of e-cigarettes have not been quantified, preliminary results suggest these products are not innocuous.

## THE PREVALENCE OF E-CIGARETTE USE IN YOUTH

E-cigarette use has become so pervasive among youth that it is impossible to go to the bathroom in many middle and high schools because so many people are “JUULing” (i.e. vaping).^43^ In response, several Massachusetts high schools have started to lock the bathrooms in order to prevent e-cigarette use during the school day.^44^ Between 2011 and 2015, e-cigarette use grew an astounding 900% among high school students. To put this into perspective, in 1976, 28.8% of high school seniors reported daily use of combustible cigarettes; by 2015, the rate had fallen to 5.6%.^19^ Recent research conducted independently in Colorado and Massachusetts reveals that 25% of high school students vape,^45^ a rate 16 times higher than that of adults.^45^ Vape products are now the most commonly used form of tobacco among youth in the United States.^44^ In response, the US Surgeon General has declared e-cigarette use among youth “an epidemic.”^46^ In a reflection of the activity’s appeal, a new term, “vape god,” has entered current lingo. A “vape god” is someone (most of the time a hipster) who is a connoisseur of electronic cigarettes (e-cigarettes) and spends all of his time and money on these products in an attempt to create “huge vape clouds in public.”^47^

The Jewish world is not immune to the e-cigarette epidemic among teens. A psychologist from Bergen County, New Jersey, USA, who works with many middle and high school students from local yeshiva day schools and high schools has stated: “Kids are JUULing in the school bathroom. Kids are JUULing in class. Teachers are now a little more aware of it, so they might be able to recognize a JUUL when they see one and know it’s not a USB device. But it’s so easy to take a hit when no one is looking, or to have it in your pocket.”^48^ The popularity of JUUL has also spread to Israeli youth; the Israel Ministry of Health considers this product “a grave risk to public health.”^49^

Eighty percent of the 15–24-year-olds who try JUUL continue using the product,^20^ and social media posts saying “addicted to my JUUL” are common.^50^ Why has vaping become so popular among youth? E-cigarettes, especially JUUL, deliver an addictive dose of nicotine without the noxious taste that often deters first-time smokers of combustible cigarettes from subsequent use. In addition, e-cigarette devices are compact and inconspicuous, resembling a USB drive, and are easy to conceal from authority figures, facilitating widespread use even in the classroom.^50^ They have a sleek, modern design, with packaging resembling a smartphone. JUULs are available in attractive-sounding flavors, including “creme,” “fruit medley,” “mango,” and “cool mint.” The packages offer misleading, kid-appealing imagery suggesting juice boxes, lollipops, and other foods, targeting youth with the illusion that these products do not contain nicotine and in fact are no more harmful than candy.^51^ Youth and young adults most commonly try e-cigarettes because of curiosity, the appeal of the flavorings, and the belief that e-cigarettes are a less harmful, less toxic alternative to conventional cigarettes. They do not turn to e-cigarettes as a way to stop smoking.^46^ In fact, youth who use e-cigarettes appear to be at a low risk of smoking conventional cigarettes; they only begin to vape because they mistakenly believe that e-cigarettes are nicotine-free and safe.^21^ Of current JUUL users aged 15–24, 64% did not know that the product always contains nicotine, nor did they recognize its effects.

## EFFICACY OF E-CIGARETTES IN SMOKING CESSATION

Manufacturers promote e-cigarettes as nicotine-replacement products that will help smokers quit smoking. In surveys, e-cigarette users believe that this product is an effective tool for quitting conventional cigarettes and reducing the risk of tobacco-related disease.^52^ American smokers trying to quit prefer these products to Food and Drug Administration (FDA)-approved cessation aids.^53^ However, the FDA has *not* approved e-cigarettes for smoking cessation.^54^ A recent study reported that after one year, the rate of abstinence from smoking tobacco was higher in the e-cigarette group (18.0%) than in the nicotine-replacement group (9.9%).^55^ The 18% abstinence rate among e-cigarette users is similar to the 20% rate of smoking cessation attained with FDA-approved medicines with acceptable safety profiles.^56^ However, 80% of the smokers who quit smoking by using e-cigarettes were still using them one year later, leading to concerns regarding the long-term safety of e-cigarette use. Thus, the argument that e-cigarette use is justified because it aids smoking cessation is invalid since FDA-approved smoking-cessation aids are no less effective.

## *HALAKHA* AND E-CIGARETTE USE

*Halakha*, or Jewish law, is the ethical code by which the traditional Jew leads his or her life. As new questions arise in Jewish law, Jewish legal scholars have historically answered them through responsa, written decisions and rulings. It is not surprising that there are no written responsa on e-cigarettes given their novelty and the relative dearth of scientific data regarding their long-term safety and efficacy in smoking cessation. Nevertheless, e-cigarettes raise several *halakhic* questions. It behooves us to begin to formulate a *halakhic* perspective on these products, especially in light of their growing popularity, and the reality that they have reached the religious world and have infiltrated yeshiva middle and high schools.

### Does *Halakha* Prohibit E-cigarette Use?

E-cigarettes contain harmful materials. However, the long-term effects of e-cigarettes are not known and might not be established for years. Is use of e-cigarettes permitted based on *shomer peta’im HaShem*, the notion that G-d watches over the simple, at least until the long-term effects are clearer, *or* do current data provide sufficient justification for prohibiting these products already?

Many of the *halakhic* principles that prohibit use of combustible cigarettes apply to e-cigarettes. Virtually all modern *halakhic* decisors prohibit smoking combustible cigarettes.^57^ Although Rav Moshe Feinstein, whom many North American Jews considered the premier modern decisor of modern Jewish law, did not do so, recently, his son, Rav Dovid Feinstein, has stated that had his father been asked today, when medical research has unequivocally confirmed the dangers of smoking, he would not have allowed it, as his dispensation was based on the “fact” that smoking endangered only a small percentage of smokers.^58^ Rabbi Moshe Tendler, professor of biology at Yeshiva University, expert in medical ethics and Rav Moshe’s son-in-law, concurs (personal communication, Rabbi Moshe Tendler, April, 2019). In fact, in a statement dated Elul 5732 (1972) and recently published in *Kovetz Hama’or*, Rav Moshe stated that if future medical data came to provide additional evidence of the dangers of smoking, indicating that the percentage of smokers who became ill had increased, he would indeed prohibit smoking.^59^

For many years, *halakhic* authorities permitted smoking combustible cigarettes based on the principle, “*shomer peta’im HaShem*,” G-d protects the simple—the notion that there are certain risky behaviors that society accepts. However, Tsitz Eliezer, Rabbi Eliezer Yehuda Waldenberg, a prolific *halakhic* decisor in Jerusalem who for the last half century addressed contemporary issues in medicine and *halakha*, states that *shomer peta’im HaShem* only applies when the risk of a behavior is unknown or undocumented, the risk is not reversible, or many people engage in this activity without harm.^60^ He cites three factors that effectively disqualify smoking from *shomer peta’im HaShem*: the publication of thousands of scientific studies establishing the dangers of smoking; the establishment of laws in multiple countries requiring warning labels on cigarette products; and the US Surgeon General’s report that smoking cessation is the most effective method of preventing lung cancer. Emphatically, he concludes that, in light of current medical data, applying *shomer peta’im HaShem* to permit smoking is absurd. Extrapolating from Tsitz Eliezer’s responsa, the documented doubled risk of myocardial infarction from e-cigarette use compared to that of non-smokers, the five-fold increase in risk when combined with combustible cigarettes, and the increased risk of COPD and emphysema all confirm the danger of these products, potentially changing them from acceptable risks to forbidden ones. Tsitz Eliezer concluded that government warnings regarding the dangers of smoking invalidate *shomer peta’im HaShem*. The FDA in the United States, the European Union, and Israel’s Ministry of Health all require e-cigarette packages to carry the label, “Warning: This product contains nicotine. Nicotine is an addictive chemical.”^61^ Although this language is not as emphatic as the warnings that appear on the packages of combustible cigarettes, it might similarly alter their *halakhic* status from permissible, based on *shomer peta’im HaShem*, to forbidden.

Some might argue that because medical data have not unequivocally established the dangers of e-cigarettes, the principle of *shomer peta’im HaShem* might theoretically permit the use of these products. Indeed, there are a number of limitations to applying Tsitz Eliezer’s prohibition against smoking combustible cigarettes to e-cigarettes and thus concluding that e-cigarettes do not have the *halakhic* status of *shomer peta’im HaShem*. First, Tsitz Eliezer and the other *poskim* who ultimately prohibited cigarette smoking in the strongest terms did not do so until many years after definitive data confirmed the dangers of combustible cigarettes and that many smokers were unable to quit.^58^,^62^ According to this reasoning, the preliminary data regarding the long-term dangers of e-cigarettes would be insufficient to create a prohibition against their use; *poskim* would do so only after a large body of evidence had accrued, confirming the effects of e-cigarettes on cardiovascular and pulmonary disease and carcinogenesis and the inability of e-cigarette users to stop using these products. However, part of the initial hesitation of *poskim* to forbid smoking cigarettes in the face of compelling scientific data attesting to the dangers of these products might have stemmed from the reality that smoking was already deeply entrenched in society when such scientific data emerged. Because e-cigarettes are a relatively new product without the popularity and acceptability of combustible cigarettes, *poskim* might be less inclined to hesitate before prohibiting e-cigarettes. In fact, they have a unique opportunity to seize this moment to stop e-cigarette use before it becomes widespread and more difficult to uproot.

Furthermore, even in the absence of extensive data regarding the long-term safety of these products, *halakha* would prohibit their use based on the reality that e-cigarettes are a gateway to combustible cigarettes, whose dangers have been sufficiently documented so that virtually all *poskim* forbid them. An additional limitation to relying on *shomer peta’im HaShem* to ban e-cigarette use is that some *poskim* define *shomer peta’im HaShem* very broadly, using the concept to justify virtually all behaviors except when the danger is immediate (such as when a rock falls on a child and we know with certainty that the child is beneath it).^58^,^62^ Since the dangers of e-cigarettes are not immediate and will not occur for many years, such *poskim* would classify these products as permitted under the principle *shomer peta’im HaShem*. However, most *poskim* reject this expansive definition.^58^,^63^ In fact, Tsitz Eliezer writes that *shomer peta’im HaShem* only applies when there is a remote suspicion of a danger that is rare.^60^ Even if more years of follow-up are necessary to establish the specific long-term dangers of e-cigarette use, the high risk that users will progress to combustible cigarettes—whose dangers are manifold and well-established—would remove e-cigarettes from the realm of *shomer peta’im HaShem*.

In addition, in Kovetz Shiurim Ketubot 136, Rav Elchanan Wasserman, a prominent rabbi in pre-World War II Europe, states that *shomer peta’im HaShem* does not apply in situations of “*minhag derech eretz*” where one is unable to protect oneself because human nature requires engaging in such behaviors. In situations that are not “*minhag derech eretz*” where one can avoid danger, *shomer peta’im HaShem* does not apply; one who does not avoid such situations will pay with his life and not be protected by heaven.^64^ E-cigarette use is a new phenomenon. It is in its early stages of popularity, not yet entrenched in society. As an avoidable behavior, it would not be likely to qualify as “*minhag derech eretz*.” Because e-cigarette use is an avoidable behavior from which one can refrain, *shomer peta’im HaShem* might not apply to such use.

Finally, even if medical literature has not firmly and definitively established the long-term dangers of e-cigarette use independent of combustible cigarettes, the suspicion that these products are dangerous is sufficient to prohibit their use. Rabbi Moses Isserles (commonly known as Rama), the author of *Mappah*, the definitive Ashkenazi gloss on *Shulchan Aruch*, writes that even a *safek sakana*, the possibility that a behavior is dangerous, creates a prohibition.^58^,^65^ If the obligation to protect one’s health is Biblical, based on the verse “*ve’nishmartem ma’od et nefshotechem*,” or “guard your health exceedingly,”^66^ then a *safek sakana* becomes a *safek de’oraita*, a case of doubt regarding a Biblical prohibition, which is even more stringent.^57^,^67^ The *halakhic* principle “*chamira sakanta meisura*”^68^—that we rule more stringently in situations of *safek* (where the outcome is uncertain) that arise from danger rather than from prohibitions—further supports a strict approach to situations where there is even a suspicion of danger.^57^ The increased risks of heart attack, COPD, and emphysema associated with e-cigarette use certainly create this *safek sakana*.

Other reasons for proscribing e-cigarettes are that they lead to nicotine addiction, which Igrot Moshe prohibits,^69^ and that they expose anyone adjacent to the smoker to second-hand smoke, an action that that Igrot Moshe and others have forbidden.^60^,^70^–^73^ Several *halakhic* authorities prohibit smoking combustible cigarettes in public places. Although Rabbi Moshe Feinstein did not prohibit smoking, he banned smoking in the *beit midrash* because second-hand smoke exposure was unpleasant for non-smokers. He held to this principle despite the possibility that his ban might force the smoker to leave the study hall and detract from his Torah learning.^70^ Several other *poskim* similarly forbid exposing others to second-hand smoke.^60^,^71^–^73^ Tsitz Eliezer 15:39 concludes that non-smokers have the privilege and right to demand that smokers refrain from smoking in their presence even if the non-smoker was previously able to tolerate the second-hand smoke.^60^ Shevet HaLevi, Rav Shmuel HaLevi Wosner, a prominent haredi decisor who lived in Bnei Brak, Israel, until his death in 2015, categorically prohibited smoking in public areas as even the smell is harmful.^71^ The 2016 US Surgeon General’s report states that e-cigarette aerosol is not harmless “water vapor,” although it generally contains fewer toxicants than combustible tobacco products.^19^ There is limited evidence on the health effects of passive vapor exposure. Passive exposure to e-cigarette vapor appears to expose bystanders to propylene glycol and glycerol, known carcinogens.^74^ Based on the opinions of Rav Moshe Feinstein and Tsitz Eliezer, it would seem that if a by-stander cannot tolerate the second-hand smoke of e-cigarettes, that in and of itself is sufficient to prohibit e-cigarette use in the presence of others. Shevet HaLevi might categorically prohibit e-cigarette use in public regardless of whether the vapors cause discomfort to bystanders.

### Are There Specific Populations for Whom *Halakhic* Authorities Might Permit E-cigarette Use?

The only population for which one might allow e-cigarettes use is that of current smokers. Two arguments might justify e-cigarette use for this group.

One could argue that a current smoker who is trying to quit is obligated to use e-cigarettes as smoking-cessation aids. However, this approach raises the *halakhic* question of whether one is permitted to substitute a potentially less harmful product, e-cigarettes, for a very harmful product, combustible cigarettes. Historically, decisors have debated the permissibility of permitting prohibited behavior to avoid a more egregious sin. For example, in the middle ages, they debated the permissibility of brothels staffed by unmarried Jewish women, allowing men to violate the minor rabbinic transgression of having sexual relations with an unmarried woman in order to avoid their transgressing the Biblical decree against having relations with a married woman. Rav Isaac Arama, a fifteenth-century Spanish rabbinic scholar and author of *Akeidat Yitshak*, refused to permit the establishment of such brothels,^75^ arguing that it is preferable for an individual to be stoned than to disregard a single letter of the Torah by permitting a prohibited behavior. Modern decisors facing contemporary questions regarding the permissibility of violating a minor prohibition in order to avoid transgressing a more severe one repeatedly invoke *Akeidat Yitshak* to argue against such behavior.^76^ Thus, we see that there is strong *halakhic* precedent for enforcing a minor prohibition even if this will lead to desecration of a major prohibition. In this view, *halakha* should not permit e-cigarette use—a less harmful, though not harmless, form of smoking—in order to avoid more dangerous and *halakhically* problematic combustible cigarettes. Even if *halakha* permitted replacing a major prohibition with a minor prohibition, the grounds for doing so in this case would be weak, as scientific evidence does not show e-cigarettes to be any more effective as smoking-cessation aids than FDA-approved products with acceptable safety profiles. The FDA does not recommend the use of e-cigarettes for smoking cessation, and most individuals who turn to them for this reason continue to use combustible cigarettes as well, exposing themselves to the dangers of both products.

A second argument for permitting current smokers to use e-cigarettes is that they are already addicted to nicotine and thus have the *halakhic* status of *onu*s—acting without free will—which consequently exempts them from a potential prohibition against e-cigarette use (the *halakhic* principle is, *onus rahmana patriah*, sin that is committed against one’s will is exempt from punishment).^57^,^77^ However, nearly 50 years before the true dangers of smoking were known, the Polish scholar, Rabbi Israel Meir HaKohen Kagan, commonly referred to as Chofetz Chaim, after the title of his book on guarding one’s tongue, unequivocally rejected the possibility that current smokers have the halakhic status of *onus rahmana patriah*, and not responsible for their actions.^78^ When smokers told him that they strongly wanted to quit but just could not stop smoking, he answered: “Whoever told you to begin smoking in the first place?” Chofetz Chaim stated that if smokers die prematurely, *HaShem* will judge them for their actions because they acted of their own free will and not out of *onus*.

### Are There Specific Populations for Whom E-cigarettes Should be Especially Prohibited?

#### Pregnant women

There is little data on the health effects of e-cigarette use in pregnancy. According to the American College of Obstetrics and Gynecology, “the quality of the available evidence is poor and well-designed trials are needed to understand the health effects of these products: their role, if any in smoking cessation, and their effects on pregnant women and their fetuses.”^79^ Because nicotine in any form poses considerable health risks and has adverse effects on fetal brain and development,^19^,^79^ e-cigarette use in pregnancy is at the very least a *safek sakana*, which Rama prohibits. Furthermore, applying Tsitz Eliezer’s conclusion that government warnings create a prohibition against smoking—with the US Surgeon General’s 2016 report stating, “Pregnant women and women intending to become pregnant should be cautioned against using e-cigarettes to avoid unnecessary nicotine exposure to their baby”^19^—classifies the use of e-cigarettes as a behavior that society is not willing to accept and that should thus be forbidden.

#### Youth

There are many reasons for prohibiting young people from using e-cigarettes. First, nicotine is particularly harmful to the developing brain. Second, e-cigarettes too often lead to the use of combustible cigarettes, which are certainly prohibited. Third, nicotine and the resulting opioid addiction violate the obligation to honor one’s parents (who most likely object to their use). Fourth, they detract from time learning, whether nicotine-addicted students feel compelled to leave the classroom to vape, or focus on how to sneak vapes during class. Fifth, Shevet HaLevi prohibits youth from smoking without exception.^71^ Finally, Tsitz Eliezer, based on his responsa on combustible cigarettes, would certainly prohibit e-cigarette use in youth in light of the US Surgeon General’s declaration that, “any e-cigarette use among young people is unsafe, even if they do not progress to future cigarette smoking.”^19^

### The US Surgeon General’s Stance and *Halakha*

In light of the added dangers of e-cigarettes in youth, the US Surgeon General’s declaration that any use of e-cigarettes in youth is unsafe, and Shevet HaLevi’s statement that it is an absolute prohibition for adolescents to initiate smoking, *do we have a unique obligation to prevent children from obtaining these products?*

Kiddushin 29a lists a father’s obligations to his son, and presents an opinion obligating him to teach his son to swim, because “his life depends on it.” Swimming lessons are potentially lifesaving. Rashi explains that if the son does not know how to swim and is on a sinking boat, he will be in danger. Although Rashi does not cite the source of this obligation, *Sefer HaChinuch*, a work published anonymously in thirteenth-century Spain that discusses each of the Torah’s 613 commandments, suggests that the obligation is based on “*lo ta’amod al dam re’echa*”^80^—do not stand idly by your neighbor’s blood—and that it requires all people (mothers included) to save anyone in danger. Expanding on this position, “*lo ta’amod al dam re’echa*” obligates a parent to save his child from other potentially life-threatening situations by instructing him in fire safety, cardiopulmonary resuscitation, careful street crossing, the importance of seatbelts, and the dangers of combustible and e-cigarettes. Although Rav Feinstein does not forbid smoking, he clearly states that a father, even one addicted to cigarettes, has a unique obligation to prevent his child from initiating smoking.^81^ Similarly, Shevet HaLevi states unequivocally that parents and teachers have a Biblical obligation to keep children from smoking.^71^ *Halakha* puts smoking prevention for children in a different category than for adults, and therefore we must take added precautions to protect children from the dangers of smoking.

### Misinformation Regarding E-cigarette Use

There is a great deal of misinformation regarding e-cigarette use. Most smokers believe that e-cigarettes aid in smoking cessation, although this is not accurate. A total of 67% of JUUL users do not realize that the product contains nicotine. Nearly half of the pregnant women who used e-cigarettes reported doing so in the belief that e-cigarettes are less harmful than regular cigarettes or that e-cigarettes would help them to stop smoking.^82^ *Are we halakhically obligated to correct society’s misperceptions regarding the safety and efficacy of e-cigarettes?*

The principle of communal responsibility is fundamental to Judaism. It obligates us to protect individuals and the larger Jewish community, and to rescue these those in danger. Sanhedrin 37a states that one who saves a single life saves an entire world. *Sefer HaChinuch*^80^ derives this obligation from “*lo ta’amod al dam re’echa*.” He states: “just as someone will save his neighbor, so too, his neighbor will save him. This is how the world will be settled as *HaShem* wants.” Shevet HaLevi states that leading rabbinic leaders must publicly decry the dangers of smoking in order to educate the public, and that one who fails to do so is guilty of *shefichat damim*, murder.^71^ The author posits that this obligation should apply to e-cigarette use as well.

As a society, we are required to inform the public, and especially its highly vulnerable populations—pregnant women and youth—of the risks of e-cigarette use. In addition to the role that parents play in informing their children of the risks of e-cigarette use, *rabbanim*, public figures, teachers, physicians, and policy makers have an obligation to educate the public about the dangers of e-cigarette use and to encourage those who are using these products to quit.

### Are Jews Permitted to Sell E-cigarettes?

Rav Moshe Feinstein states that lighting a cigarette for a smoker does not violate “*lifnei iver*,”^83^,^84^ the prohibition against causing another Jew to violate the Torah, suggesting that he would not prohibit a Jew from selling e-cigarettes. However, his son Rabbi Dovid Feinstein, believes that if asked today, with our current understanding of the dangers of smoking, Rav Moshe would strongly prohibit smoking and any efforts that enable it.^58^ Given the known health risks of cigarettes, Shevet HaLevi states that anyone who has the ability to stop someone else from smoking must do so and that this obligation is Biblical.^71^

From the author’s perspective, the sale of e-cigarette products to children violates the prohibition of “*lifnei iver*.” We have a unique *halakhic* obligation to prevent young people from starting to smoke because use of these products is particularly dangerous and is prohibited by the US Surgeon General, and because the sale of e-cigarettes to minors is illegal. In an effort to prevent youth from using e-cigarettes, the FDA has proposed limiting the sale of flavored e-cigarette products to age-restricted, in-person locations, increasing age verification for online sales, and banning e-cigarette products that are marketed to children or youth through popular children’s cartoons, animated characters, or product names that appeal to children such as brands of candy or soda.^51^ In the USA, the states of Illinois, California, Hawaii, Maine, Massachusetts, New Jersey, Oregon, and Virginia have all raised the legal age for smoking (including vaping) to 21 in an effort to keep minors from using e-cigarettes.^85^

### Is a Jewish Society Permitted to Allow the Advertising of E-cigarettes?

Rambam, also known as Maimonides, the medieval Sephardic Jewish philosopher and physician whom many regard as the most influential Torah scholar of the Middle Ages, states: “It is forbidden to mislead people in business or to ‘steal their mind.’” This applies equally to gentiles and Jews. Thus, if one knows of a product defect, he must inform the purchaser. According to Rambam, it is even forbidden to deceive people with words only (i.e. where no loss of money results).^86^ Given the known health risks of cigarette smoking, Shevet HaLevi has ruled that newspapers may not advertise cigarettes.^71^ A popular advertisement for JUUL that appears throughout the city of Ra’annana, Israel, where the author resides, proclaims that the product does not contain “tobacco leaves.” It is true that JUUL does not actually contain tobacco leaves, but it does contain tobacco. The advertisement deliberately misleads consumers, leading them to think that the product is nicotine-free. This is an outright misrepresentation and is exactly what Rambam defines as “deception with words.” The US Surgeon General recommends that communities “implement strategies to curb e-cigarette advertising and marketing that are appealing to young people.”^19^ This includes prohibiting “child-friendly” advertisements, displaying advertisements near schools, and marketing campaigns aimed at minors. It is incumbent upon the Jewish community not to allow this kind of advertising.

## MODERN *POSKIM*

Rav Professor Avraham Steinberg, director of the Medical Ethics Unit at Shaare Zedek Medical Center, Jerusalem, and author of the *Encyclopedia of Jewish Medical Ethics*, the most comprehensive textbook ever compiled on the subject, agrees with the author’s assessment regarding both the prohibition against e-cigarette use, especially by youth and pregnant women, and the sale and advertisement of these products (personal communication, Rav Avraham Steinberg, April 2019). Rabbi Moses Tendler stated that were Rav Feinstein alive today, when we recognize the true dangers of cigarettes, he would certainly prohibit use of combustible cigarettes on a Biblical level. Regarding the *halakhic* permissibility of e-cigarettes, Rabbi Tendler believes that their safety is irrelevant, as their addictive nature, destroying the free will of those who use them, renders them prohibited on a Biblical level. He said that if scientific evidence shows that e-cigarettes are a more effective aid to smoking cessation than existing products, current smokers would not only be permitted to use these products, but would be obligated to do so. However, we as a society must take special precautions to ensure that youth do not have indiscriminate access to these products. The only qualification to this ban would be the case where a psychologist or counselor prescribes e-cigarettes as a way to help the adolescent stop smoking. Similarly, while selling these products is prohibited Biblically (as it is forbidden for one to endanger his friend), there might be an exception if the purchaser can provide documentation, such as a prescription from a healthcare provider, confirming that he is purchasing e-cigarettes for a therapeutic purpose such as smoking cessation (personal communication, Rabbi Moses Tendler, April 2019).

## CONCLUSION

E-cigarettes are a relatively new product whose long-term risks we do not yet fully understand. Nevertheless, their popularity, particularly among youth, is undeniable. Although they might be less harmful than combustible cigarettes, e-cigarettes are certainly not harmless, especially for pregnant women as nicotine exposure can harm the fetus, and youth, who are at high risk for nicotine addiction and for whom they all too easily lead to the use of combustible cigarettes. Few modern *poskim* have weighed in on the permissibility or *halakhic* issues surrounding e-cigarette use. However, extrapolations from *halakhic* discussions regarding the use of combustible cigarettes suggest that e-cigarettes should be forbidden, in particular for pregnant women and young people, populations for whom the dangers are amplified and to whom governments have issued warnings against cigarette use. We are obligated as a society to educate the public regarding the dangers of e-cigarettes, to prevent children from obtaining e-cigarettes, and to halt the false advertising and perhaps even the sale of e-cigarettes, especially to youth.

## Abbreviations

COPD: chronic obstructive pulmonary disease
FDA: US Food and Drug Administration.
